# Direct application of Padé approximant for solving nonlinear differential equations

**DOI:** 10.1186/2193-1801-3-563

**Published:** 2014-09-27

**Authors:** Hector Vazquez-Leal, Brahim Benhammouda, Uriel Filobello-Nino, Arturo Sarmiento-Reyes, Victor Manuel Jimenez-Fernandez, Jose Luis Garcia-Gervacio, Jesus Huerta-Chua, Luis Javier Morales-Mendoza, Mario Gonzalez-Lee

**Affiliations:** Electronic Instrumentation and Atmospheric Sciences School, Universidad Veracruzana, Cto. Gonzalo Aguirre Beltrán S/N, 91000 Xalapa, Mexico; Higher Colleges of Technology, Abu Dhabi Men’s College, P.O. Box 25035, Abu Dhabi, United Arab Emirates; National Institute for Astrophysics, Optics and Electronics, Luis Enrique Erro No. 1, Sta. Maria, 72840 Tonantzintla, Puebla México; Dirección General de la Unidad de Estudios de Posgrado, Universidad Veracruzana, Av. Paseo de las Palmas No. 15 esq. Los Mangos, Fracc. Jardines de las Ánimas, 91000 Xalapa, Veracruz México; Facultad de Ingenieria Civil, Universidad Veracruzana, Venustiano Carranza S/N, Col. Revolucion, 93390 Poza Rica, Veracruz México; Department of Electronics Engineering, Universidad Veracruzana, Venustiano Carranza S/N, Col. Revolucion, 93390 Poza Rica, Veracruz México

**Keywords:** Padé transform, Nonlinear differential equations

## Abstract

**Abstract:**

This work presents a direct procedure to apply Padé method to find approximate solutions for nonlinear differential equations. Moreover, we present some cases study showing the strength of the method to generate highly accurate rational approximate solutions compared to other semi-analytical methods. The type of tested nonlinear equations are: a highly nonlinear boundary value problem, a differential-algebraic oscillator problem, and an asymptotic problem. The high accurate handy approximations obtained by the direct application of Padé method shows the high potential if the proposed scheme to approximate a wide variety of problems. What is more, the direct application of the Padé approximant aids to avoid the previous application of an approximative method like Taylor series method, homotopy perturbation method, Adomian Decomposition method, homotopy analysis method, variational iteration method, among others, as tools to obtain a power series solutions to post-treat with the Padé approximant.

**AMS Subject Classification:**

34L30

## 1 Introduction

Solving differential equations is an important issue in sciences because many physical phenomena are modelled using such equations. The Padé method is a well established resummation method from literature. It can increase the domain of convergence of truncate power series (Bararnia et al. 2012; Guerrero et al. 2013; Torabi and Yaghoobi 2011; Vazquez-Leal and Guerrero 2013). It is has been applied to the improve the accuracy of truncated power obtained by power series method (PSM) (Forsyth 1906; Geddes 1979; Ince 1956; Vazquez-Leal and Guerrero 2013), Adomian Decomposition method (ADM) (Wazwaz 2006; Wang et al. 2011), homotopy perturbation method (HPM) (Bararnia et al. 2012; Rashidi and Keimanesh 2010; Torabi and Yaghoobi 2011), homotopy analysis method (HAM) (Guerrero et al. 2013), differential transform method (DTM) (Rashidi and Keimanesh 2010; Rashidi et al. 2010; Rashidi and Pour 2010a, 2010b), among others, during the solution procedure for linear and nonlinear differential equations. Nonetheless, in this work, we propose that the solution of a differential equation can be directly expressed as a rational power series of the independent variable, in other words as a Padé approximant. The proposed procedure will be described by solving several nonlinear problems and comparing results with other semi-analytic methods. The direct application of Padé eradicates the necessity to obtain a power series solution (by some approximative method) to post-treat it with the Padé approximant. Instead, we substitute a Padé approximant of a given order directly to the nonlinear differential equation; it results a residual power series in terms of the independent variable. Next, from the lowest order, we equate each coefficient of such power series to zero, resulting a system of nonlinear algebraic equations (NAEs). Finally, we resolve the NAEs in order to minimize the residual error of the differential equation.

This paper is organized as follows. In Section 2, we introduce the basic concepts of the Padé approximant. Next, the procedure to approximate nonlinear differential equations with Padé is presented in Section 3. In Section 4 some cases study are presented. In Section 5, numerical simulations and a discussion about the results are provided. Finally, a brief conclusion is given in Section 6.

## 2 Padé approximant

Given an analytical function *u*(*t*) with Maclaurin’s expansion
1

The Padé approximant to *u*(*t*) of order [*L*,*M*] which we denote by [*L*/*M*]_*u*_(*t*) is defined by (Baker 1975)
2

where we considered *q*_0_ = 1, and the numerator and denominator have no common factors.

The numerator and the denominator in (2) are constructed so that *u*(*t*) and [*L*/*M*]_*u*_(*t*) and their derivatives agree at *t* = 0 up to *L* + *M*. That is
3

From (3), we have
4

From (4), we get the following systems
5

and
6

From (5), we calculate first all the coefficients *q*_*i*_,1 ≤ *i* ≤ *M*. Then, we determine the coefficient *p*_*i*_,0 ≤ *i* ≤ *L* from (6).

Note that for a fixed value of *L* + *M* + 1, the error (3) is smallest when the numerator and denominator of (2) have the same degree or when the numerator has degree one higher than the denominator.

## 3 Padé applied to solve nonlinear differential equations

It can be considered that a nonlinear differential equation can be expressed as
7

having as boundary condition
8

where *L*_1_ and *N*, are a linear and a non-linear operator, respectively; *B* is a boundary operator, *Γ* is the boundary of domain *Ω*, and *∂u*/*∂η* denotes differentiation along the normal drawn outwards from *Ω*.

Now, we assume that the solution for (7) can be written as
9

where *v*_0_,*v*_1_,… and *w*_0_,*w*_1_,… are unknowns to be determined by the Padé method, *L*, *M* are the order of the numerator and denominator, and *x*_0_ is an arbitrary constant.

There is not a systematic method to choose the optimal Padé order [*L*/*M*] for a given problem. However, usually, a finite number of terms are required in order to obtain a highly accurate Padé approximation. The basic process of direct Padé procedure can be described as:

1. The boundary conditions of (7) are substituted in (9) to generate an equation for each boundary condition. It is important to notice, that there is an algebraic equation for each boundary condition, hence, the rest of equations required to generate a NAEs (with the same number of variables and equations) are obtained from the next step.
2. *u*(*x*) from (9) is substituted into (7), then, we regroup the resulting equation in terms of the *x*-powers. It is important to notice that the operators *L*_1_ and *N* will be applied to *u*(*x*). After this, the regrouping procedure will include the eradication of the denominator terms emanated from the Padé approximant (9). In this way, the resulting expression is a power series that represents the residual error of the differential equation (7).
3. In order to reduce the residual error; from the lowest order, we equate each coefficient of the *x*-powers in the resulting residual power series to zero to obtain an algebraic equation in terms of the unknown coefficients of (9).
4. Aforementioned steps generates a NAEs in terms of the unknowns from (9).
5. Finally, we solve the NAEs to obtain *v*_0_,*v*_1_,… and *w*_0_,*w*_1_,….

## 4 Cases study

In this section, we will solve several nonlinear problems of different types to show the validity and power of the direct application of Padé method to solve a broad spectrum of equations.

### 4.1 A boundary value problem

The Troesch’s equation is a boundary value problem (BVP) derived from research on the confinement of a plasma column by radiation pressure (Weibel 1959) and also from the theory of gas porous electrodes (Gidaspow and Baker 1973; Markin et al. 1966). The problem is expressed as
10

where prime denotes differentiation with respect to *x* and *n* is known as Troesch’s parameter.

In order to facilitate the application of Padé method, we convert the hyperbolic-type nonlinearity from Troesch’s problem into a polynomial type nonlinearity (Chang 2010; Vazquez-Leal et al. 2012c), using the variable transformation
11

After using (11), we obtain the following transformed problem
12

where conditions are obtained by using variable transformation (11).

Then, substituting original boundary conditions *y*(0) = 0 and *y* (1) = 1 into (11), results
13

We suppose that solution for (12) has the following rational expression
14

where *w*_0_ = 1, *x*_0_ = 0, and *L* = *M* = 8.

Substituting (14) into (12), rearranging and equating terms having the same *x*-powers, we obtain
15

Next, equating coefficients of *x* in (15) to zero, we obtain the following system of nonlinear algebraic equations
16

Now, in order to consider the boundary conditions (13), we substitute them into (14) to obtain
17

corresponding to *u* (0) = 0 and , respectively.

Then, solving the system composed by (16) and (17), it results
18

and
19

for *n* = 0.5 and *n* = 1, respectively.

Finally, from (11) and (14), the proposed solution of Troesch’s problem is
20

where (18) or (19) are used depending on the value of *n*.

### 4.2 Differential-algebraic equation

Consider the index one differential-algebraic equation system (DAEs) (Amat et al. 2012)
21

where prime denotes derivative with respect to *t*, and the exact solution is
22

We suppose that solution for (21) has the following rational form
23

where *w*_1,0_ and *w*_2,0_ are considered as 1 to simplify the process of solution, and *t*_0_ = 0.

If we consider *L*_1_ = *M*_1_ = *L*_2_ = *M*_2_ = 12, and substituting (23) into (21); rearranging and equating terms having the same *t*-powers, we obtain
24

Next, equating coefficients of *t* in (24) to zero, we obtain the following system of nonlinear algebraic equations
25

Now, in order to consider the initial conditions from (21), we substitute them into (23) to obtain
26

corresponding to  and , respectively.

Solving the NAEs composed by (25) and (26), it results the coefficients shown in Table 1.

**Table 1:** **Coefficients from Padé approximant (**27**) for DAEs (**21**)**

***i***	***v***_1,***i***_	***w***_1,***i***_	***v***_2,***i***_	***w***_2,***i***_
0	0.7071067812	1	0.7071067812	1
1	0.6944478949	-0.01790236873	-0.710785391	-0.005202339869
2	-0.3552705766	0.015473901	-0.3381563291	0.01657239326
3	-0.1007688245	-0.0002670569686	0.1079008196	-0.0001006409133
4	0.02599872528	0.0001213625776	0.02316102487	0.0001405811897
5	0.003720336719	-1.958219599e-06	-0.004157250847	-9.478457134e-07
6	-0.0006440541915	6.292610474e-07	-0.0005234980593	7.978499480e-07
7	-5.257637767e-05	-9.004870369e-09	6.112387339e-05	-5.582330983e-09
8	6.857858371e-06	2.327029094e-09	4.879283240e-06	3.277470106e-09
9	3.140311387e-07	-2.662291749e-11	-3.785438873e-07	-2.120465882e-11
10	-3.286935291e-08	5.983862645e-12	-1.897258525e-08	9.536760323e-12
11	-6.790217810e-10	-4.175830173e-14	8.457510263e-10	-4.313868912e-14
12	5.933734260e-11	8.658872139e-15	2.337878489e-11	1.599248188e-14

From (23) and Table 1, the proposed solution is
27

### 4.3 Asymptotic problem

The quadratic Riccati equation is a well known, and difficult to solve, asymptotic problem for approximative methods (Abbasbandy 2006, 2007; Tan and Abbasbandy 2008; Tsai and Chen 2010). The problem is expressed as follows
28

where prime denotes differentiation with respect to *t*. The exact solution of (28), was found to be
29

We suppose that solution for (28) has the following rational form
30

where *w*_0_ = 1, *t*_0_ = 0, and *L* = *M* = 4.

Substituting (30) into (28), rearranging and equating terms having the same *t*-powers, we obtain the following system of equations
31

In order to consider the initial condition of *Y* (0) = 0, we substitute it into (30) to obtain
32

Then, using (32) and (31) and solving, results
33

Now, we will obtain another Padé approximant from (29), for the expansion point *Y* (1.7) = 2.28577828560. Therefore, using such point as initial condition, we generate the following extra equation
34

Next, using (31) and (34) to obtain the coefficients from Padé expression (30), and substituting *t* by expansion point (*t*-1.7), results

[!b]
35

**Figure 1:**
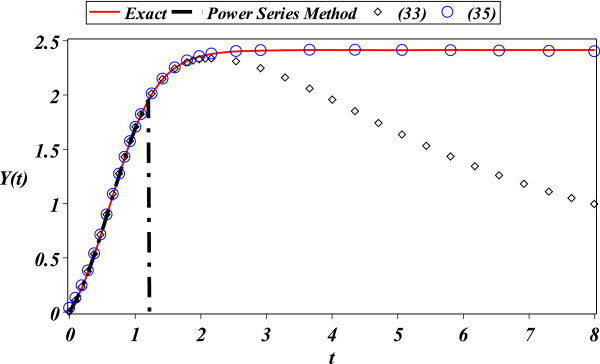
**Exact solution (**29**) (solid line), Padé approximations (**33**) (diamonds), (**35**) (circles), and a 250 terms power series solution (dash-dot).**

We can see in Figure 1 a comparison between (33) and (35) to exact solution (29). It results that changing the expansion point was useful to increase the domain of convergence of the Padé method for this case study. However, a systematic procedure to choose the optimal expansion point for general problems is a pending task for future research.

## 5 Numerical simulation and discussion

On one side, semi-analytic methods like: generalized homotopy method (GHM) (Vazquez-Leal 2013), homotopy perturbation method (Araghi and Rezapour 2011; Araghi and Sotoodeh 2012; Bayat et al. 2013, 2014; Biazar and Eslami 2011; Biazar and Ghanbari 2012; Filobello-Nino et al. 2012a, 2012b; He 1999, 2009; Khan et al. 2012a, 2012b; Vazquez-Leal 2012; Vazquez-Leal et al. 2012a, 2012b, 2012d), homotopy analysis method (Hassana and El-Tawil 2011; He 2004; Rashidi *et al*2012a,2012b; Tan and Abbasbandy^2008^), variational iteration method (Abbasbandy^2007^; Chang 2010; Khan et al. 2012c), among others (Khan et al. 2012d), need an initial approximation for the sought solutions and the calculus of one or several adjustment parameters. If the initial approximation is properly chosen, the results can be highly accurate, nonetheless, there is not a general method to choose such initial approximation. This issue motivates the use of adjustment parameters obtained by minimizing the least-squares error with respect to the numerical solution. On the other side, the Padé method obtain its coefficients using a straightforward procedure. Furthermore, at least for low-order approximations, the solution can be easily obtained using the “solve” or “fsolve” commands of MAPLE or equivalent routines from Mathematica or MATLAB.

We presented several cases study to show the successful use of the Padé method to solve directly a wide variety of nonlinear problems. For instance, the Troesch’s BVP problem is a benchmark equation for numerical (Erdogan and Ozis 2011; Lin et al. 2008) and semi-analytical methods (Chang 2010; Deeba 2000; Feng et al. 2007; Hassana and El-Tawil 2011; Khuri 2003; Mirmoradia et al. 2009; Vazquez-Leal et al. 2012c) due to the numerical problems to solve it. Nevertheless, as shown in Table 2, the Padé approximation (20) is exact for *n*=0.5 compared to the numerical solution reported in (Erdogan and Ozis 2011; Lin et al. 2008). This result is relevant considering the high error values of the solutions reported using other semi-analytical methods: homotopy perturbation method (HPM) (Feng et al. 2007; Mirmoradia et al. 2009; Vazquez-Leal et al. 2012c), Adomian Decomposition method (ADM) (Deeba et al. 2000), homotopy analysis method (HAM) (Hassana and El-Tawil 2011) and Laplace decomposition transform method (LDTM) (Khuri 2003). All of them possess an average absolute relative error (A.A.R.E.) significantly larger that our results. A similar result was found for *n*=1 as presented in Table 3. Therefore, the direct Padé method can, potentially, be an excellent tool to solve nonlinear BVP problems described over finite intervals. It is important to remark that for boundary conditions over finite intervals, the traditional Padé approximant applied to the power series of the exact solution or to the exact solution, can only guarantee one boundary condition (traditionally at *x*=0). However, the proposed method build a restriction equation for each non-singular boundary conditions over the finite interval. Such equations are part of the NAEs that is resolved to provide the coefficients of the Padé approximant. Therefore, the resulting modified Padé expression fulfils all the boundary conditions.

**Table 2:** **Comparison between (**20**), exact solution (Erdogan and Ozis** 2011 **; Lin et al.** 2008 **), and other reported approximate solutions**

***x***	Exact	This work	HPM	ADM	HPM	HPM	HAM	LDTM
	(Erdogan and Ozis 2011; Lin et al. 2008)	(20)	(Vazquez-Leal et al. 2012c)	(Deeba et al. 2000)	(Feng et al. 2007)	(Mirmoradia et al. 2009)	(Hassana and El-Tawil 2011)	(Khuri 2003)
0.1	0.0959443493	0.0959443493	0.0959443155	0.0959383534	0.0959395656	0.095948026	0.0959446190	0.0959443520
0.2	0.1921287477	0.1921287477	0.1921286848	0.1921180592	0.1921193244	0.192135797	0.1921292845	0.1921287539
0.3	0.2887944009	0.2887944009	0.2887943176	0.2887803297	0.2887806940	0.288804238	0.2887952148	0.2887944107
0.4	0.3861848464	0.3861848464	0.3861847539	0.3861687095	0.3861675428	0.386196642	0.3861859313	0.3861848612
0.5	0.4845471647	0.4845471647	0.4845470753	0.4845302901	0.4845274183	0.4845599	0.4845485110	0.4845471832
0.6	0.5841332484	0.5841332484	0.5841331729	0.5841169798	0.5841127822	0.584145785	0.5841348222	0.5841332650
0.7	0.6852011483	0.6852011483	0.6852010943	0.6851868451	0.6851822495	0.685212297	0.6852028604	0.6852011675
0.8	0.7880165227	0.7880165227	0.7880164925	0.7880055691	0.7880018367	0.788025104	0.7880181729	0.7880165463
0.9	0.8928542161	0.8928542161	0.8928542059	0.8928480234	0.8928462193	0.892859085	0.8928553997	0.8928542363
	Order	[12/12]	2	6	2	2	6	3
	A.A.R.E.	0	1.83327e(-07)	3.47802e(-05)	3.57932e(-05)	2.44418e(-05)	2.51374e(-06)	3.10957e(-08)

Calculated for *n* = 0.5.

**Table 3:** **Comparison between (**20**), exact solution (Erdogan and Ozis** 2011 **; Lin et al.** 2008 **), and other reported approximate solutions**

***x***	Exact	This work	HPM	ADM	HPM	HPM	HAM	LDTM
	(Erdogan and Ozis 2011)	(20)	(Vazquez-Leal et al. 2012c)	(Deeba et al. 2000)	(Feng et al. 2007)	(Mirmoradia et al. 2009)	(Hassana and El-Tawil 2011)	(Khuri 2003)
0.1	0.0846612565	0.0846612565	0.08466075858	0.084248760	0.0843817004	0.084934415	0.0846732692	0.08466308972
0.2	0.1701713582	0.1701713582	0.1701704581	0.169430700	0.1696207644	0.170697546	0.1701954538	0.1701750442
0.3	0.2573939080	0.2573939081	0.2573927827	0.256414500	0.2565929224	0.258133224	0.2574302342	0.2573994845
0.4	0.3472228551	0.3472228551	0.3472217324	0.346085720	0.3462107378	0.348116627	0.3472715981	0.3472303763
0.5	0.4405998351	0.4405998352	0.4405989511	0.439401985	0.4394422743	0.44157274	0.4406610140	0.4406093753
0.6	0.5385343980	0.5385343981	0.5385339413	0.537365700	0.5373300622	0.539498234	0.5386072529	0.5385460046
0.7	0.6421286091	0.6421286092	0.6421286573	0.641083800	0.6410104651	0.642987984	0.7526899495	0.6421421393
0.8	0.7526080939	0.7526080940	0.7526085475	0.751788000	0.7517335467	0.753267551	0.7526899495	0.7526226886
0.9	0.8713625196	0.8713625198	0.8713630450	0.870908700	0.8708835371	0.871733059	0.8714249118	0.8713748860
	Order	[12/12]	2	6	2	2	6	3
	A.A.R.E.	1.46588e(-10)	2.54568e(-06)	0.002714577	0.002320107	0.002044737	0.019244326	2.05e(-05)

Calculated for n = 1.

**Figure 2:**
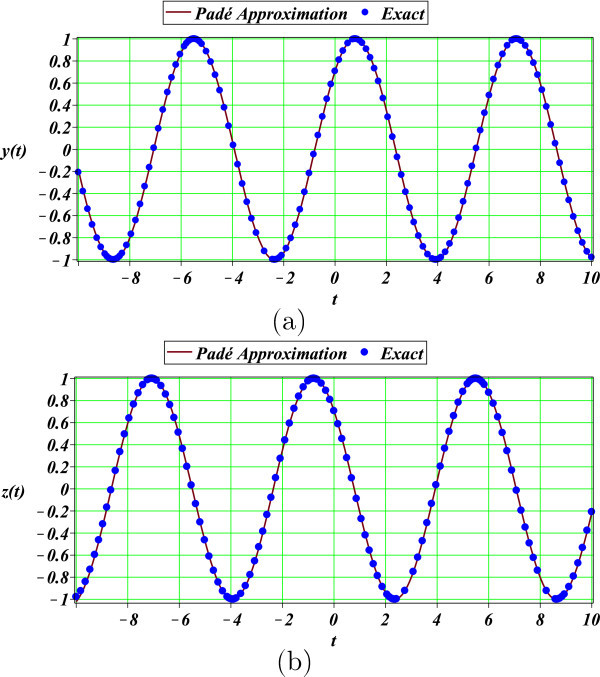
**Exact solution (**22**) (solid circles) of DAEs (**21**) and Padé approximation (**27**) (solid line): a)** ***y*** **(** ***t*** **) and b)** ***z*** **(** ***t*** **).**

Padé approximation (27) of DAEs problem (21) exhibited highly accurate results for a long period of time as depicted in Figure 2 and Table 4. The differential-algebraic nonlinear problems are of relevance on several fields of science, including microelectronics and chemistry. In addition, there is not any standard analytical method to solve this type of equations, this is what it makes the Padé method in an attractive tool to obtain approximate solutions for DAEs problems. Furthermore, the solution procedure of (21) shows that is possible - potentially - to approximate a wide variety of problems containing several variables.

**Table 4:** **Relative error (R.E.) of exact solution (**22**) versus Padé approximation (**27**)**

***t***	Exact***y***(***t***)	Exact***z***(***t***)	R.E.***y***(***t***)of (27)	R.E.***z***(***t***)of (27)
-10	-0.2086321515	-0.9779941847	0.09330825406	0.09330825406
-9	-0.9356781623	-0.3528546112	0.002548752664	0.002548752664
-8	-0.8024659858	0.5966978646	0.0002522953745	0.0002522953745
-7	0.0685297173	0.9976490755	0.0001597267828	0.0001597267828
-6	0.8765195143	0.4813663272	3.803747675e-07	3.803747675e-07
-5	0.8786413122	-0.4774824024	5.388747443e-09	5.388747443e-09
-4	0.0729443397	-0.9973360132	3.131783529e-10	3.131783529e-10
-3	-0.7998173223	-0.6002434930	2.587566848e-14	2.587566848e-14
-2	-0.9372306267	0.3487101265	9.922514670e-19	9.922514670e-19
-1	-0.2129584152	0.9770612639	1.391674918e-25	1.391674918e-25
0	0.7071067812	0.7071067812	0.0000000000	0.0000000000
1	0.9770612639	-0.2129584152	2.900333665e-26	2.900333665e-26
2	0.3487101265	-0.9372306267	2.436171789e-18	2.436171789e-18
3	-0.6002434930	-0.7998173223	3.003755589e-14	3.003755589e-14
4	-0.9973360132	0.0729443397	1.897834931e-11	1.897834931e-11
5	-0.4774824024	0.8786413122	7.783182416e-09	7.783182416e-09
6	0.4813663272	0.8765195143	5.122538684e-07	5.122538684e-07
7	0.9976490755	0.0685297173	7.591788287e-06	7.591788287e-06
8	0.5966978646	-0.8024659858	0.0002175967642	0.0002175967642
9	-0.3528546112	-0.9356781623	0.003968289586	0.003968289586
10	-0.9779941847	-0.2086321515	0.01052925646	0.01052925646

The accuracy of approximations (33) and (35) for the quadratic Riccati problem (28) is depicted in Figure 1. Moreover, we have suggested a strategy to increase the domain of convergence of the Padé method by changing its expansion point. As depicted in Figure 1, the approximation (35) obtained by expanding at *t* = 1.7 is far more accurate than (33) obtained by expanding at *t* = 1.7. It is important to notice that the expansion point was arbitrary choose for this case study; therefore, further work is required to deduce a systematic algorithm to choose optimal expansion points. Furthermore, in (Abbasbandy 2006) was reported a power series solution for the same equation with poor convergence, making necessary to solve the problem by a multi-stage version of HPM method. The advantage of our solution, in this case, is that we do not need to use a complicated segmented method; therefore, this approach generates simpler solutions. In addition, in (Tsai and Chen 2010) was reported the combination of Laplace Adomian Decomposition Method with Padé (LADM-Padé) of order [13/12] to obtain a similar result to our [4/4] order Padé solution. Furthermore, in (Abbasbandy 2007) was reported a power series solutions with short domain of convergence. A HAM solution in terms of exponential expressions was reported in (Tan and Abbasbandy 2008), presenting a high accurate solution with a larger domain than the proposed solution; for this case, we can increase the order of the Padé approximation to obtain a good agreement with HAM solution. Moreover, in order to show the advantage of the proposed method, we calculated 250 terms of the power series solution using the well established series method (using the command *dsolve* of Maple 16), resulting a poor region of convergence, followed by (33). Finally, as depicted in Figure 1, the best domain of convergence was obtained from the Padé approximant (35) due to the expansion point change.

The direct application of the Padé approximant to obtain rational solutions of nonlinear differential equations circumvent the old requirement of using Taylor series method (Vazquez-Leal et al. 2014), HPM, VIM, HAM, DTM, PSM, ADM and others, as tools to obtain a power series solutions to post-process later by the application Padé approximant. Therefore, this new straightforward methodology reduce the computational effort producing good results.

In general terms, we know from literature (Bararnia et al. 2012; Guerrero et al.^2013^; Torabi and Yaghoobi 2011; Vazquez-Leal and Guerrero 2013) that larger values for *M* and *L*, can lead to better results for Padé approximant, this considering that we count in advance with a suitable power series (large enough) obtained using an extra approximative method as aforementioned. Then, our proposal has a strong advantage because we do not require a power series to post-process with Padé approximant, because the method consist in the direct application of Padé. However, a systematic procedure to obtain the optimal order [*L*/*M*] is still a pending issue to study in a future research derived from this paper. Finally, in the present study, we restricted the research to nonsingular initial conditions and Dirichlet finite interval boundary conditions; nonetheless, further work is required to deal with singular initial condition problems, Neumann boundary conditions, infinity boundary conditions, among others.

## 6 Conclusions

This work presented the direct application of Padé method as a technique with high potential to solve nonlinear differential equations. Also, a comparison between the results of applying the proposed procedure and other semi-analytical was shown. The results showed that Padé is a powerful method to solve different nonlinear equations like the ones for: boundary value problems, differential-algebraic problems, and asymptotic problems. The method provided better results than many of the most used methods like: HPM, ADM, HAM, DTM, VIM, PSM, among others. Finally, further research should be performed to solve other kind of problems as: nonlinear fractional/partial differential equations, Pantograph equations, among others.
